# Effect of Jardiance on glucose uptake into astrocytomas

**DOI:** 10.1007/s11060-024-04746-8

**Published:** 2024-07-22

**Authors:** Chiara Ghezzi, Benjamin M. Ellingson, Albert Lai, Jie Liu, Jorge R. Barrio, Ernest M. Wright

**Affiliations:** 1 Department of Physiology, The David Geffen School of Medicine at UCLA, Los Angeles, CA 90095-1751 USA; 2 Department of Radiological Sciences, The David Geffen School of Medicine at UCLA, Los Angeles, CA 90095 USA; 3 Department of Neurology, The David Geffen School of Medicine at UCLA, Los Angeles, CA 90095 USA; 4 Department of Molecular and Medical Pharmacology, The David Geffen School of Medicine at UCLA, Los Angeles, CA 90095 USA

**Keywords:** SGLT2, SGLT2i, PET, Me4FDG, Glioblastoma

## Abstract

**Purpose:**

SGLT2, the sodium glucose cotransporter two, is expressed in human pancreatic, prostate and brain tumors, and in a mouse cancer model SGLT2 inhibitors reduce tumor glucose uptake and growth. In this study we have measured the effect of a specific SGLT2 inhibitor, Jardiance® (Empagliflozin), on glucose uptake into astrocytomas in patients.

**Methods:**

We have used a specific SGLT glucose tracer, α-methyl-4-[^18^F]fluoro-4-deoxy-α-D-glucopyranoside (Me4FDG), and Positron Emission Tomography (PET) to measure glucose uptake. Four of five patients enrolled had WHO grade IV glioblastomas, and one had a low grade WHO Grade II astrocytoma. Two dynamic brain PET scans were conducted on each patient, one before and one after treatment with a single oral dose of Jardiance, a specific SGLT2 inhibitor. As a control, we also determined the effect of oral Jardiance on renal SGLT2 activity.

**Results:**

In all five patients an oral dose (25 or 100 mg) of Jardiance reduced Me4FDG tumor accumulation, highly significant inhibition in four, and inhibited SGLT2 activity in the kidney.

**Conclusions:**

These initial experiments show that SGLT2 is a functional glucose transporter in astocytomas, and Jardiance inhibited glucose uptake, a drug approved by the FDA to treat type 2 diabetes mellitus (T2DM), heart failure, and renal failure. We suggest that clinical trials be initiated to determine whether Jardiance reduces astrocytoma growth in patients.

**Supplementary Information:**

The online version contains supplementary material available at 10.1007/s11060-024-04746-8.

## Introduction

Glucose is required for the metabolism and growth of tumors. Facilitated glucose transporters, GLUTs, or sodium glucose cotransporters, SGLTs, are responsible for glucose uptake into cells (see [1]). SGLTs are expressed in epithelial cells while GLUTs are widely expressed in cells throughout the body. SGLT2, sodium glucose cotransporter two, is largely restricted to the renal cortex where it plays a major role in the reabsorption of glucose filtered by the kidneys [2]. Highly specific SGLT2 inhibitors have been developed and approved by the FDA to treat type 2 diabetes mellitus (T2DM), and they lower blood glucose by excreting glucose into the urine (see [3]). Previously, we have reported that SGLT2 is expressed in pancreatic, prostate, and brain tumors [4, 5], and furthermore reported that SGLT2 inhibitors reduced the uptake of a specific SGLT glucose tracer, α-methyl-4-[^18^F]fluoro-4-deoxy-α-D-glucopyranoside (Me4FDG), into tumors grown in nude mice and reduced growth [4].

Me4FDG is not a substrate for GLUT transporters, and it has been used as a radiotracer for in vivo SGLT activity using Positron Emission Tomography (PET) in both mice and man [5–7]. We have shown that Me4FDG is accumulated in glioblastomas and that SGLT2 protein is expressed in the tumors and the blood-tumor-barrier (BTB). SGLT2 is not expressed in the blood-brain-barrier (BBB), and so Me4FDG does not enter the brain of control subjects [5, 6, 8]. Given that highly specific SGLT2 inhibitors, SGLT2i, have been successfully introduced to treat T2DM and heart failure [3, 9], it has been natural to speculate about the use of SGLT2 inhibitors as a cancer therapy [10]. Indeed, cancer outcomes in diabetes patients treated with SGLT2 drugs have begun to appear (see [11]).

In this study we have set out to determine the functional activity of SGLT2 in astrocytomas in patients. Our approach was to use PET to measure Me4FDG uptake into these tumors before and after treatment with a Jardiance®, a high affinity, specific SGLT2 inhibitor (SGLT2i). Patients were given a single oral dose of Jardiance 2–4 h before the second PET scan. The efficiency of Jardiance in inhibiting SGLT2 under this protocol was evaluated by measuring the excretion of Me4FDG into the urinary bladder.

While we would have preferred to carry our study on newly diagnosed patients as in our 2018 study [5], the standard of care does not permit two experimental PET scans before surgery. Instead, we chose to conduct our experiments on four patients with grade IV recurrent tumors after initial surgical resection, radiation, and chemotherapy, and one with a grade II astrocytoma (Table 1). In all five cases Jardiance reduced the uptake of Me4FDG. We conclude that our results suggest a basis for conducting clinical trials to determine whether Jardiance and other SGLT2 inhibitors can reduce the growth of these devastating tumors.

**Table 1:** Astrocytoma patients

PATIENT age*	DIAGNOSIS	Location	Extent of Resection	INTERVAL (months) From diagnosis to PET	Prior Treatment
EP7 male 61	Glioblastoma, IDH-wildtype, CNS WHO grade 4	Bi-frontal midline	Sub-total resection	9	RT, Temozolomide, Avastin
JP8 male 34	Low grade diffuse astrocytoma, H3 K27M WT, CNS WHO Grade II	Left cerebellar, brainstem.	Biopsy	66	RT, Temozolomide
JP9 male 62	Glioblastoma, IDH-wildtype, CNS WHO grade 4	Left temporal-parietal.	Gross total resection	5	RT, Temozolomide
SP11 male 52	Glioblastoma, IDH-wildtype, CNS WHO grade 4	Left parietal lobe	Sub-total resection	25	RT, Temozolomide
DP12 male 66	Glioblastoma, IDH-wildtype, CNS WHO grade 4	Left temporal lobe	Sub-total resection	6	RT, Temozolomide

• Age at diagnosis

The diagnosis, location, resection, the time interval between original diagnosis and Me4FDG PET study, and the prior treatment of five male patients. RT radiation therapy

## Methods

### Subjects

Patients with recurrent tumors were referred to one of us (AL) for evaluation, and five suitable ones were enrolled (Table 1) after providing their written informed consent. Our study was performed in compliance with the guidelines established by the UCLA Institutional Review Board for human experiments and the UCLA Jonsson Comprehensive Cancer Center. Each patient was evaluated using MRI imaging, (T1-weighed MP-RAGE with and without gadolinium contrast), as part of their clinical care, and then subjected to two MeF4DG PET studies two to ten days apart (Table S1). Patient 1 (EP7) is a 61-year-old male with a bi-frontal, heterogeneously enhancing recurrent glioblastoma (IDH wild type, EGFR amplified) that previously failed radiation, temozolomide and bevacizumab treatments. The patient has irregular and nodular enhancement surrounding the resection cavity, with tumor extending across the genu of the corpus callous, resulting in significant thickening and midline shift with areas of mild restricted diffusion and elevated cerebral blood volume on perfusion MRI. Slight mass effect was observed on the lateral ventricles. Patient 2 (JP8) is a 34-year-old male with an IDH wild type grade II astrocytoma in the cerebellum and brain stem exhibiting heterogeneous enhancement and expansile T2/FLAIR hyperintense signal abnormality after failure of temozolomide rechallenge. Patient 3 (JP9) is a 62-year-old male patient with a ring enhancing left temporal lobe newly diagnosed glioblastoma with unknown molecular characteristics receiving adjuvant temozolomide following gross total resection and concurrent radiation and temozolomide. Patient 4 (SP11) is a 52-year-old male with a left parietal IDH wild type recurrent glioblastoma contiguous with the left posterior lateral ventricle that previously failed concurrent radiation and temozolomide. Patient 5 (DP12) is a 66-year-old male with an IDH wild type recurrent glioblastoma in the left temporal lobe and previously failed concurrent radiation and temozolomide.

### PET imaging

Imaging was conducted as described previously in the UCLA Ahmanson Nuclear Medicine Clinic [5]. Me4FDG was prepared by nucleophilic fluorination of methyl 2,3,6-tri-O-acetyl-4-O-triflyl-α-D-galactopyranoside using cyclotron-produced [^18^F-]fluoride (revised from [12] and to be published elsewhere). The carrier free product, > 97% radiochemical pure, with a specific activity > 2,000 Ci/mmol. was added to 154 mM NaCl and filtered through a sterile filter for injection into subjects. Approximately 370 MBq (10 mCi) was injected as a bolus through an indwelling venous catheter (Table S1) and the scan was conducted on a Siemens Biograph/CTI ECAT scanner. Immediately after Me4FDG injection a 30-minute dynamic brain scan was conducted, and this was followed by a whole-body scan from the top of the head to mid-thigh consisting of seven bed positions for 3-minutes each. The whole-body scan was used to determine the amount of Me4FDG excreted into the urinary bladder. Attenuation correction was obtained from a one-minute CT scan prior to the PET scan. The acquired PET data covering 15.5-cm in the axial field of view was reconstructed using the filtered-back projection method (after correction for attenuation, dead time, scatter, and isotope decay) with a Hanning filter into 128 × 128 × 63 matrices. The spatial resolution of brain and whole-body images were ∼ 2.5 and ∼ 5.5 mm in full width at half maximum. The PET images were analyzed using AMIDE software [13], and activities are given in MBq/gram. 3D regions of interest (ROI) were placed over the tumor, on brain regions remote from the tumor to determine brain background (BG), and the torcula to determine blood activity (Input function). Preliminary experiments established that Input Functions determined from PET images compared well with those obtained from arterialized blood samples (see Phelps 2004). The maximum Me4FDG uptake at each time point of the dynamic scans was obtained from the tumor voxels with > 90 of the activity.

Analysis of Me4FDG uptake into the tumor is expressed by the ratio of the tumor ROI to the torcula ROI (SUVR) and the signal (S) to noise (N) ratio for the tumor relative to the background (BG) for white and grey matter. Tumor voxels with at least 90% of the maximum value within the tumor ROI were used to calculate the peak uptake (SUVR_peak_). Values are given as the mean with the standard error of the mean, and N the number of estimates. The significance of differences between means before and after treatment with Jardiance were evaluated by unpaired t-tests to obtain *p*-values (Table S2).

Fusion of MRI and PET scans were performed using previously described techniques [14–16]. First, DICOM images were converted to the Neuroimaging Informatics Technology Initiative (NIFTI) standardized format using *dcm2niix* (https://github.com/rordenlab/dcm2niix). Next, all PET images were linearly registered (6 degrees of freedom) to pre-treatment, post-contrast T1-weighted MRI scans, using *FLIRT* (https://fsl.fmrib.ox.ac.uk/fsl/fslwiki/FLIRT), the image registration tool from FSL (FMRIB Software Library; http://www.fmrib.ox.ac.uk/fsl), an open-source and freely available library of analysis tools for brain [17].

Following evaluation of the first PET scan, the second Me4FDG PET scan was conducted within ten days after a single oral dose of Jardiance, 25 or 100 mg: 25 mg /day is the FDA approved dose for treatment of T2DM, and 100 mg produces the maximum 24-hour renal glucose excretion in control subjects [18]. Jardiance^R^ (empagliflozin 25 mg tablets, Boehringer Ingelheim / Lilly) obtained from the Pharmacy at the UCLA Medical Center. Tablets were ingested by subjects 2–4 h prior to the Me4FDG PET scan (Table S1). The iso-contour method was used to align tumor uptakes before and after treatment with Jardiance. No adverse side effects of the single oral dose of Jardiance or the two PET scans were encountered.

## Results

**Fig. 1:**
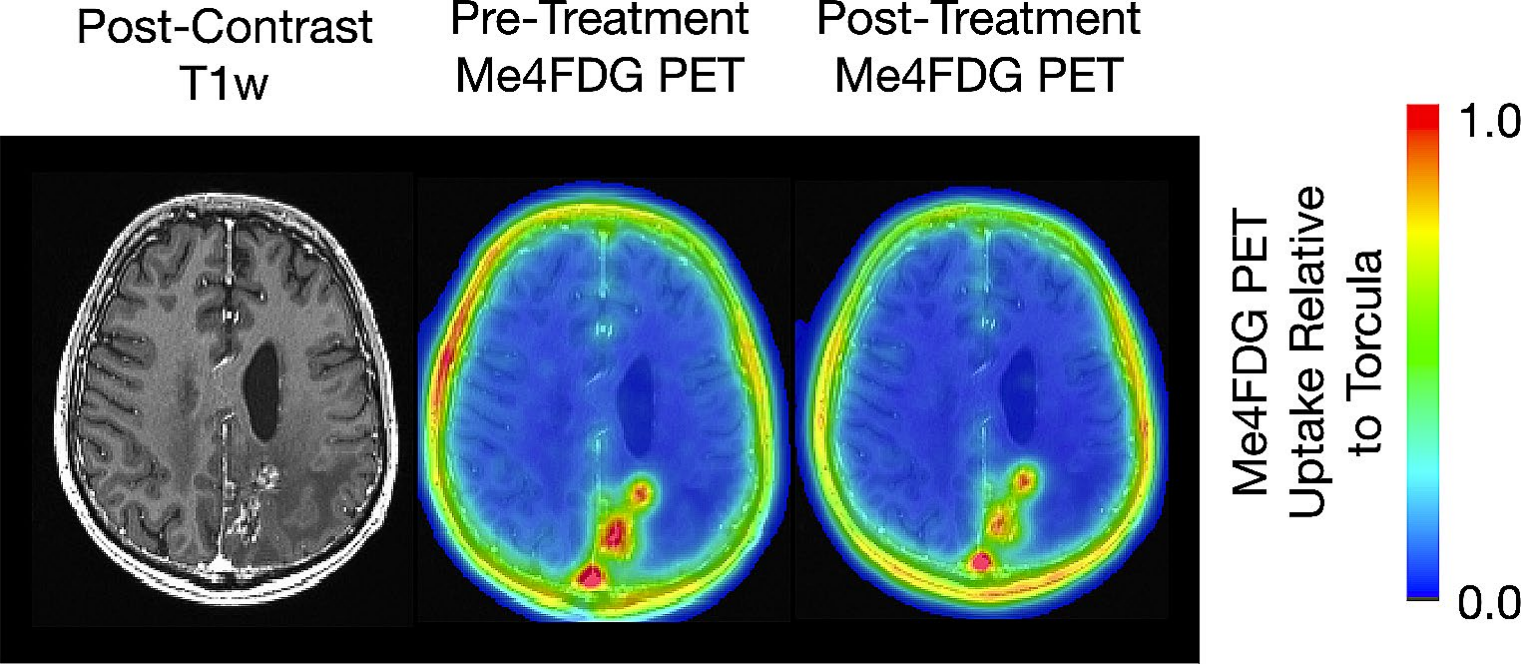
Me4FDG PET scans and a post-contrast T1w MRI of a 52-year-old male patient (SP11) with a recurrent left parietal lobe WHO grade IV glioblastoma. The PET scans were conducted before and after treatment with an oral 100 mg dose of Jardiance. Both the Me4FDG PET scans and the MRI also show the left parietal mass, 4.9 × 3.3 × 4.1 cm, and a second smaller lesion in the left thalamus. The PET images were taken from the sum of the last three frames between 15 and 30 min (see Fig. 2A). No malignancies were detected in whole body CT and Me4FDG PET images

Figure 1 shows trans-axial brain MRI and Me4FDG PET sections of a 52-year-old male patient (SP11) with a recurrent left parietal lobe WHO grade IV glioblastoma and a secondary lesion in the left thalamus. Despite standard of care therapy, including sub-total resection, radiation, and chemotherapy, recurrent tumor was detected within 25 months. The post-contrast T1w MRI image shows the location of the major tumor resection, and the recurrent tumor growth around the resection cavity. The Me4FDG PET images, pre- and post-treatment with Jardiance, show Me4FDG accumulation corresponding to the areas of gadolinium uptake, especially around the rim of the resection cavity. There was no accumulation Me4FDG in any other region of the brain apart from the tumors, but there was accumulation in the extracranial temporalis muscles as noted previously [5]. After a single oral dose of 100 mg Jardiance there was a visible reduction of Me4FDG uptake into the tumors and the extracranial muscles.

**Fig. 2:**
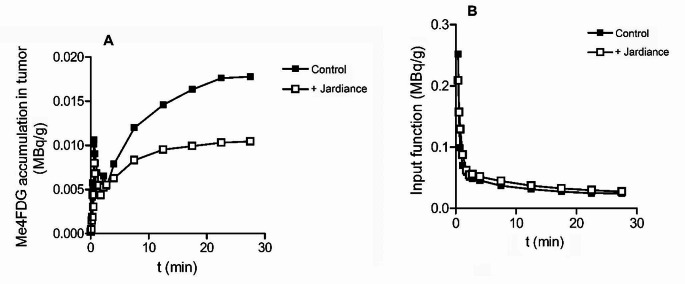
The time course of Me4FDG accumulation in the left parietal lobe tumor before and after oral treatment with Jardiance. (**A**) The two PET scans on patient SP11 were conducted seven days apart, and 100 mg of Jardiance was taken 190 min before the beginning of the second PET scan. Jardiance increased the excretion of Me4FDG into the urinary bladder from 7 to 33 MBq, or from 2 to 10% of the injected dose. (**B**) The Input Functions, the blood activity, for these two Me4FDG PET scans using ROIs placed over the Torcula (confluence of the brain sinuses). In the five subjects there was no significant difference between the Input Functions in the absence and presence of Jardiance

The time course of Me4FDG uptakes into the left parietal tumor before and after Jardiance treatment is shown in Fig. 2A. After an initial transient peak due to the distribution of the injected dose of Me4FDG throughout the vascular tree, Me4FDG accumulated in the tumor to a steady state value in the absence of Jardiance of 0.017 MBq/g with a half-time of ∼ 8 min. Treatment with 100 mg Jardiance significantly reduced the uptake to 0.010 MBq/g with no apparent change in the half-time. Figure 2B shows the time course of the Input Function, the activity of Me4FDG in the Torcula, the venous blood draining the brain. The initial peak at ∼ 0.5 min is due to the distribution of the tracer in the vascular tree after i.v. injection, and the decay is due to the distribution of Me4FDG from blood into the extracellular and intracellular compartments throughout the body. It is notable that Jardiance treatment did not alter the Input Function or the background (BG) for white and grey matter. Under our protocol Jardiance inhibited renal SGLT2 as recorded by the excretion of Me4FDG into the urinary bladder. On average 0.5% of the injected Me4FDG dose (10 mCi or 370 MBq) was excreted into the bladder in one hour, while after the oral dose of Jardiance 10% was excreted (see Figure legends).

Analysis of Me4FDG uptake into the tumor is expressed by the ratio of the tumor ROI to the torcula ROI (SUVR) and the signal (S) to noise (N) for the tumor relative to the background (BG) for white and grey matter (Table S2). Tumor voxels with at least 90% of the maximum value within the tumor ROI were used to calculate the peak uptake (SUVR_peak_) 1.30 *±* 0.08 (N 3) before and 0.83 *±* 0.10 (N 3) after treatment with Jardiance (p 0.003). The S/N ratio was 7.18 *±* 0.32 (N 3) before and 4.38 *±* 0.23 (N 3) after Jardiance p < (0.001).

The brain background values (BG) were 365 vs. 341 Bq/g, in the absence and presence of Jardiance and this confirmed that there are no functional SGLTs, specifically SGLT2, in the normal blood-brain-barrier (BBB). The S/N ratio for white/gray matter remote from the tumor was 0.09 (N 3)[5]. For the extracranial temporalis muscle, the SUVR_peak_ was 1.47+-0.03 (N 3) before and 1.21 *±* 0.03 (N 3) after Jardiance demonstrating that SGLT2 contributes to glucose uptake into this muscle [5]

**Fig. 3:**
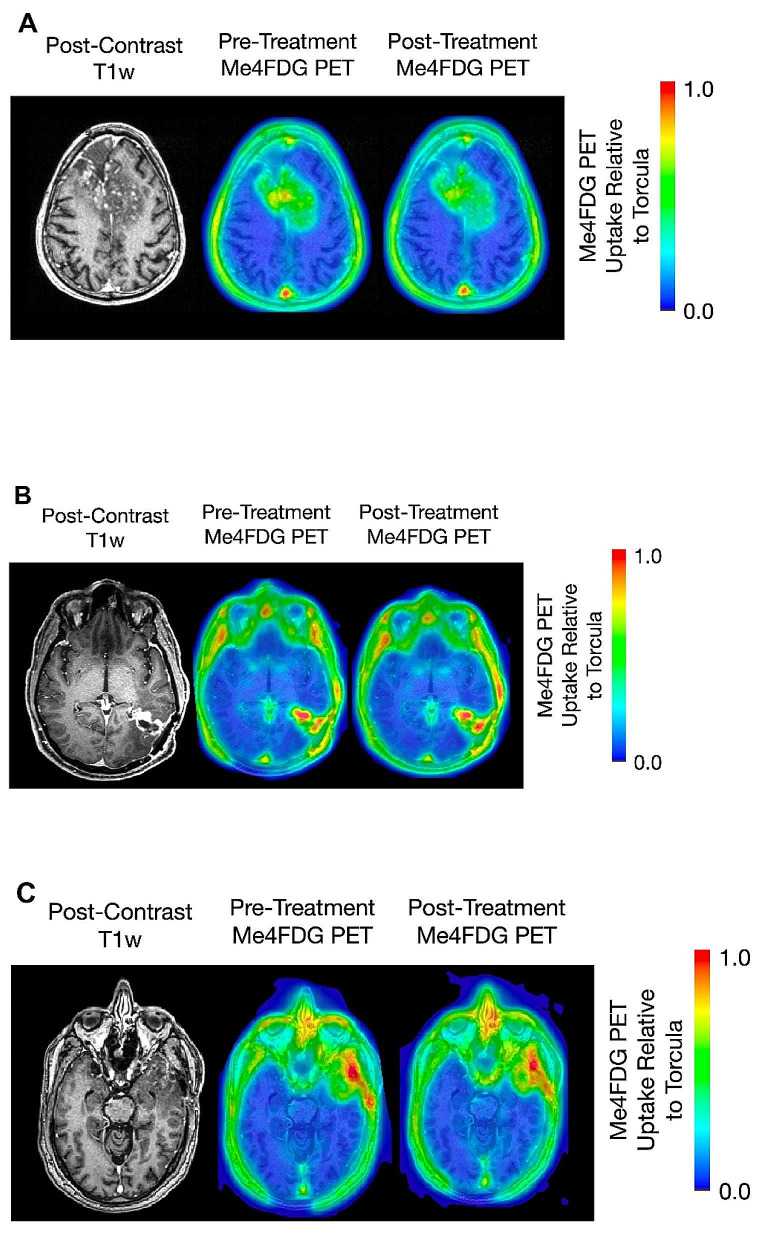
Me4FDG PET and MRI brain scans of three WHO grade IV astrocytoma patients. **A**. E7P with a bi-frontal midline tumor, **B.** J9P with a left temporal parietal tumor, and **C**. D12P with a left temporal lobe tumor. See Fig. 1 for details. Patients EP7 and JP9 were treated with 25 mg of Jardiance that resulted in an increase of Me4FDG renal excretion from 0.5 to 14 and 12% of the injected dose, while patient DP12 was treated with 100 mg that resulted in an increase in renal Me4FDG excretion from 0.5 to 8% of the injected dose

The results for the three other patients with recurrent WHO grade IV tumors are shown in Fig. 3, MRI and Me4FDG PET images (A, B, C), and in Figure S1, the time courses for Me4FDG uptakes (A, B, C): (A) a 61-yr old patient (EP7) with a bifrontal midline WHO stage IV tumor was studied 9 months after diagnosis; (B) a 62-year old patient (JP9) with left temporal parietal lobe WHO IV tumor who was studied 5 months after diagnosis; and (C) a 66-year old patient (DP12) with left temporal lobe WHO stage IV tumor who was studied 6 months after diagnosis. In all cases Me4FDG was only accumulated in the tumors, SUVR_p_ > 1.0, and treatment with Jardiance visually reduced Me4FDG uptake into the tumors (see also Table S2) and the extracranial muscles. In each case the uptake of Me4FDG coincides with the location of gadolinium in the MRI, and treatment with Jardiance reduced the uptake (Fig. 3): The SUVRp values were reduced from 1.58 *±* 0.03 (N 3) to 1.34 *±* 0.06 (N 3), 1.16 *±* 0.02 (N 3) to 0.94 *±* 0.03 (N 3), and 1.18 *±* 0.08 (N 3) to 1.09 *±* 0.02 (N 3) Table S2).

**Fig. 4:**
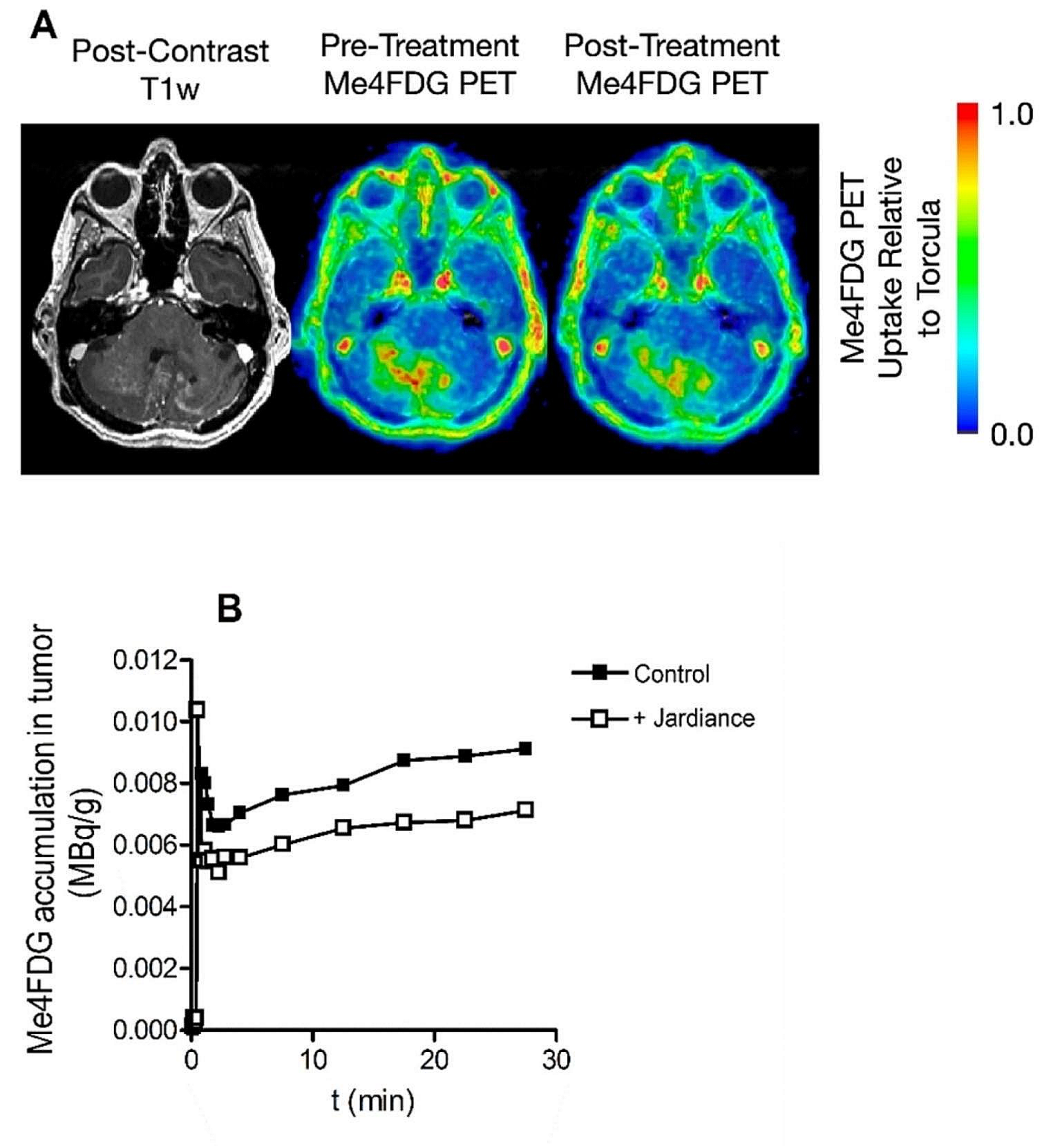
**A**. Me4FDG PET scans and a post-contrast T1w MRI of a 34-year-old male patient (JP8) with a diffuse cerebellar brainstem WHO grade II astrocytoma. **B**. The time courses of Me4FDG accumulation this tumor IV before and after an oral 25 mg dose of Jardiance. The uptake parameters are given in Table S2. This dose of Jardiance increased the renal excretion of Me4FDG from 1 to 3.5% of the injected dose in one hour

Finally, the results for a 34-year-old patient (JP8) with a diffuse brainstem WHO grade II astrocytoma are shown in Fig. 4. The uptake of Me4FDG into focal regions of the tumor were also reduced by Jardiance, (Fig. 4A), as was the time course of uptake (Fig. 4B). There was a 5–8% decrease in the SUVR_p_ and S/N ratios, from 1.17 *±* 0.05 (n 3) and 3.69 *±* 0.12 (n 3) to 0.93 *±* 0.07 (n 3) and 2.99 *±* 0.24 (n 3) respectively (Table S2).

## Discussion

This study uses a specific SGLT radiotracer and PET imaging to show that glucose uptake into low-grade and high-grade gliomas, WHO grade II and IV, is mediated at least in part by SGLT2. Me4FDG is a specific, non-metabolized tracer for sodium glucose transporters that is not a substrate for GLUTs [6, 7, 19]. SGLTs, couple the uphill transport of glucose into cells to the sodium electrochemical potential gradient across the cell membrane. The SGLT2 isoform is normally restricted to the kidney proximal tubule where it is largely responsible for the reabsorption of glucose from the glomerular filtrate [2, 7, 19]. The kidneys filter about 180 g of glucose from plasma each day but less than 0.5 g are voided in the urine and SGLT2 is responsible for ∼ 50% of the kidney glucose reabsorption. SGLT2 drugs such as Jardiance^R^ block this component [18].

We have previously reported that SGLT2 is expressed in glioblastomas [5]. Namely, we have demonstrated that Me4FDG is accumulated in high grade astrocytomas using PET, and that SGLT2 protein is expressed in neoplastic cells and the endothelium lining tumor microvasculature of the glioblastomas. Here we extend this work to show that Jardiance^R^, (empagliflozin), a highly potent and specific inhibitor of SGLT2 activity in cells and in vivo [9], inhibits Me4FDG uptake into both high-grade and low-grade tumors (Figs. 1, 2, 3 and 4). The effectiveness of oral doses of Jardiance in our patients is confirmed by (i) the increase in excretion of Me4FDG into the urinary bladder and (ii) the inhibition of Me4FDG uptake into extracranial muscles.

The significance of GLUTs in glucose uptake into patient tumors remains unknown despite our comparison of 2-[^18^F]fluoro-2-deoxy-D-glucose (2FDG) and Me4FDG uptakes in high grade astrocytomas [5]. The reason being that the accumulation of 2FDG depends on GLUT expression AND intracellular phosphorylation, while non-metabolized Me4FDG accumulation only depends on SGLT2 activity [8]. Our only clue is that SGLT2 plays a more important role than GLUTs in maintaining viable tumor cells is that an SGLT2i reduces tumor growth and increases necrosis in a mouse pancreatic xenograph model [4].

Questions must be raised about how Me4FDG and Jardiance gain access to tumors in patients, Namely, SGLT2 is not expressed in the blood-brain-barrier (BBB) as documented by the fact that Me4FDG does not enter the brain from blood in rodents and man [5, 6, 8] and Figs. 1 and 3, and 4), and that SGLT2 protein is not detected in the BBB [5]. Osmotic opening of the BBB permits Me4FDG to enter the rodent brain [8]. MRI studies with gadolinium contrast agents typically show contrast enhancing of high-grade tumors, e.g., Figs. 1 and 3, indicating vascular leakage across the blood-tumor barrier (BTB). Contrast enhancement is not normally observed for low-grade tumors but is apparent in the one case studied (Fig. 4A). It is speculated that gadolinium (MW 567) leaks through the tight junctions of the endothelium of the BTB. Similar access to the extracellular compartment of tumors across the BTB may occur for Me4FDG (MW 196) and Jardiance (MW 451). It is possible that Me4FDG could be transported into tumor extracellular spaces by SGLT2 expressed in the endothelium of the proliferating microvascular of high-grade tumors [5], but this alone would not account for accumulation of Me4FDG within tumors cells (SUVR > 1).

Following our original report showing that SGLT2 is expressed and functional in pancreatic and prostrate tumors isolated from patients, SGLT2 inhibitors reduced Me4FDG uptakes into pancreatic and prostate cells grown in nude mice [4], a wealth of literature has followed raising the potential use of SGLT2 inhibitors as anticancer agents [11, 20–23]. Those studies that have exposed cancer patients to SGLT2 drugs indeed suggest that their addition to the standard of care improves the prognosis of a variety of cancer patients. Indeed, it is timely to initiate clinical trials of SGLT2 inhibitors on cancer patients, including those with astrocytomas, especially given the high affinity, specificity and safely of this class of drugs.

### Electronic supplementary material

Below is the link to the electronic supplementary material.

Supplementary Material 1

## Data Availability

The data generated are available from the corresponding author on reasonable request.
