# Supplementary material for: Effect of Jardiance on glucose uptake into astrocytomas
Source: J Neurooncol. 2024 Jul 22;169(2):437–44. doi: 10.1007/s11060-024-04746-8 (PMC11341586; doi:10.1007/s11060-024-04746-8)
Supplement: Supplementary file 1 — Supplementary Material 1 [file 11060_2024_4746_MOESM1_ESM.docx]

**SUPPLEMENTARY MATERIAL**

**Effect of Jardiance on Glucose Uptake into Astrocytomas**

Chiara Ghezzi^1^, Benjamin M. Ellingson^2^, Albert Lai^3^, Jie Liu^4^, Jorge R. Barrio^4^, Ernest M Wright^1*^

The SM contains one Figure (S1) showing the time course of Me4FDg accumulation in tumors before and after treatment with Jardiance, and two Tables: Table S1 summarizing the study of five patients: and Table S2 showing the uptake of Me4FDG into tumors before and after Jardiance.


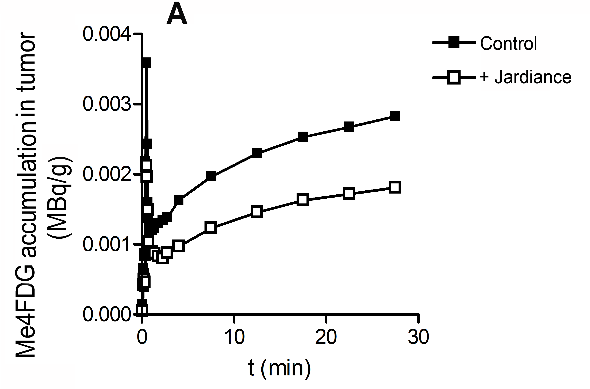






**Figure S1. The time courses of Me4FDG accumulation in WHO stage IV tumors before and after an oral dose of Jardiance (see Figure 3).** **A.** EP7 with a bi-frontal midline tumor, **B**. J9P with a left temporal parietal tumor, and **C.** DP12 with a left temporal lobe tumor (see Tables 1 and S2, and Figure 3). EP7 and JP9 were given a 25 mg oral dose and DP12 a 100 mg oral dose of Jardiance 2-4 hours before PET scan.

**TABLE S1. Me4FDG PET Study of Table 1 astrocytoma patients.**

| **Patient** | **EP7** | **JP8** | **JP9** | **SP11** | **DP12** |
| --- | --- | --- | --- | --- | --- |
| Age | 61 | 34 | 62 | 52 | 66 |
| Weight | 158 | 183 | 198 | 120 | 165 |
| **First PET** | 7.17.2019 | 8.15.2019 | 9.18.2019 | 2.9.2023 | 2.13.2023 |
| Me4FDG | 374 MBq | 303 MBq | 359 MB) | 303 MBq | 394 MBq |
| Glucose | n.d. | 83 mg% | 87 mg% | 91 mg% | 79 mg% |
| **Second PET** | 7.25.2019 | 8.20.2019 | 9.20.2019 | 2.16.2023 | 2.23.2023 |
| Me4FDG | 370 MBq | 300 MBq | 366 MBq | 381 MBq | 374 MB |
| Glucose | 117 mg% | 92 mg% | 88 mg% | 74 mg% | 78 mg% |
| Jardiance | 25mg | 25 mg | 25 mg | 100 mg | 100 mg |
| Time between Jardiance and PET | 199 min | 133 min | 183 min | 190 min | 166 min |

Patient data, including age (at diagnosis), weight, blood glucose, and time interval between oral Jardiance treatment and initiation of the Me4FDG PET for each patient. The study was interrupted by the COVID19 pandemic.

**TABLE S2 Me4FDG uptake into tumors before and after Jardiance**

|  | **SUVR_p_** Tumor vs Torcula | | | |  | **S/N Ratio** | | | | |
| --- | --- | --- | --- | --- | --- | --- | --- | --- | --- | --- |
|  | **Before*** | SEM | **Afte**r | SEM | p | **Before** | SEM | **After** | SEM | p |
| **EP7** | 1.16 | 0.02 | 0.94 | 0.03 | 0.001 | 5.22 | 0.14 | 4.42 | 0.23 | 0.007 |
| **JP8** | 1.17 | 0.05 | 0.93 | 0.07 | 0.008 | 3.69 | 0.12 | 2.99 | 0.24 | 0.01 |
| **JP9** | 1.19 | 0.08 | 1.09 | 0.02 | 0.08 | 6.07 | 0.34 | 5.75 | 0.12 | 0.2 |
| **SP11** | 1.30 | 0.08 | 0.83 | 0.10 | 0.003 | 7.18 | 0.32 | 4.38 | 0.23 | <0.001 |
| **DP1**2 | 1.59 | 0.03 | 1.34 | 0.06 | 0.003 | 6.69 | 0.11 | 5.22 | 0.26 | 0.001 |

For each patient the mean SUVR_peak_ and S/N ratios were obtained from three regions of interest with voxels having >90% of Me4FDG activity. *Previous SUVR_peak_ 2.03, S/N 13.9 [ Kepe, V., et al., *Positron emission tomography of sodium glucose cotransport activity in high grade astrocytomas.* J Neurooncol, 2018. **138**(3): p. 557-569] [5].
